# Oryeongsan suppressed high glucose-induced mesangial fibrosis

**DOI:** 10.1186/s12906-015-0542-6

**Published:** 2015-02-22

**Authors:** Jung Joo Yoon, Yun Jung Lee, So Min Lee, Dae Gill Kang, Ho Sub Lee

**Affiliations:** Professional Graduate School of Oriental Medicine and College of Oriental Medicine, Wonkwang University, Shinyong-dong, Iksan, Jeonbuk 570-749 Republic of Korea; Hanbang Body-fluid Research Center, Wonkwang University, Shinyong-dong, Iksan, Jeonbuk 570-749 Republic of Korea; Brain Korea (BK) 21 plus team, Professional Graduate School of Oriental Medicine, Wonkwang University, Iksan, Jeonbuk 540-749 Republic of Korea

**Keywords:** Oryeongsan, Mesangial cell, Proliferation, Fibrosis

## Abstract

**Background:**

The pathological change of kidney in diabetic nephropathy is represented hypertrophy, inflammation, and renal fibrosis. Oryeongsan, traditional oriental herbal formula, is widely used for the treatment of nephrosis, dropsy, and uremia. This study was examined whether Oryeongsan attenuate high-glucose (HG)-promoted rat mesangial cell fibrosis and matrix accumulation, major features of diabetic glomerulosclerosis.

**Methods:**

Oryeongsan was mixed traditional herbal medicine, *Alisma orientale* Juz, *Polyporus umbellatus* Fries, *Atractylodes macrocephala* Koidez, *Poria cocos* Wolf and *Cinnamomum Cassia* Presl (5:3:3:1). Renoprotective role in diabetic nephropathy of Oryeongsan was evaluated by [^3^H]-thymidine incorporation, Western blot, RT-qPCR and immunofluorescence microscopy assay.

**Results:**

Rat mesangial cell proliferation induced by HG was significantly accelerated, which was inhibited by Oryeongsan in a dose dependent manner. HG enhanced expression of fibrosis biomarkers such as collagen IV and connective tissue growth factor (CTGF), which was markedly attenuated by Oryeongsan. Oryeongsan increased HG-inhibited membrane type-1 matrix metalloproteinase expression (MT1-MMP) and MMP-2 promotor activity, whereas suppressed HG-induced tissue inhibitor of matrix metalloproteinase-2 (TIMP-2) expression. Moreover, Oryeongsan promoted extracellular matrix degradation through disturbing transforming growth factor β (TGF-β)–Smad signaling. This study further revealed that Oryeongsan ameliorated HG-induced mesangial inflammation accompanying induction of intracellular cell adhesion molecule-1 (ICAM-1) and monocyte chemoattractant protein-1 (MCP-1). Moreover, pretreatment of Oryeongsan inhibited NF-κB translocation in HG-exposed mesangial cell.

**Conclusion:**

These results demonstrate that Oryeongsan has protective effect against renal proliferation, fibrosis, and inflammation. Therefore Oryeongsan may be specific therapies targeting renal dysfunction leading to diabetic nephropathy.

## Background

Diabetic nephropathy is characterized by aberrant alterations such as extracellular matrix (ECM) accumulation ultimately leading to chronic renal failure. The pathological changes of diabetic nephropathy include kidney hypertrophy, glomerulus and tubular basement membrane thickening, tubular interstitial fibrosis and arteriosclerosis.

Increased mesangial cell proliferation is one of the major pathologic features in the early stage of diabetic nephropathy [1]. Knowledge of the role of rat mesangial cells in normal glomeruli and of their response to pathological stimuli is crucial to the understanding of these disease processes [2]. There are few data on the effects of pharmacological intervention on mesangial cell proliferation in diabetic nephropathy.

The first and most distinctive glomerular lesion of diabetes is mesangial expansion concurrently accompanying mesangial hyperplasia, which precedes interstitial disease called diabetic glomerulosclerosis [3]. Mesangial cells, which are contractile, smooth muscle-like cells located in the intercapillary space of the glomerular tufts, are thought to be the primary producers of mesangial ECM constituents. The mesangial matrix is normally composed of various macromolecules, including fibronectin, laminin, collagen, and thrombospondin, as well as various proteoglycans [4]. Mesangial cells are thought to play an important role in the metabolism of type IV collagen controlling its synthesis and degradation, increased synthesis or decreased degradation of type IV collagen by mesangial cells could result in the expansion of ECM, leading to mesangial lesion expansion [5].

CTGF may contribute to diabetic renal disease not only by induction of ECM synthesis but also through inhibition of matrix degradation. HG caused a decrease in ECM degradation by mesangial cells, and in glomeruli [6]. This lesser degradation has been shown to occur through changes in the balance of the family of metal ion-dependent enzymes known as the matrix metalloproteinases (MMPs) and their specific inhibitors, the tissue inhibitors of MMPs (TIMPs), in particular, TIMP-1. We have recently shown that CTGF mediates the effects of HG to inhibit human renal mesangial cell matrix degradation through the up-regulation of TIMP-1 by CTGF [7].

TGF-β1 is thought to play an important role in mediating the hypertrophic and fibrotic/sclerotic manifestations of diabetic nephropathy [8]. Previous studies have provided evidence that TGF-β1 mediates the accumulation of ECM molecules in mesangial cells and tubular cells [9]. Inhibition of TGF-β1 significantly reduced renal fibrosis and decreased the mRNA levels of key mediators of ECM deposition in the kidneys of db/db mouse [10]. Thereby, induction and activation of the TGF-β/Smad signaling cascade is critical for the initiation of fibrogenic cell responses. Subsequently, ligand binding causes a recruitment and phosphorylation of receptor-Smads (R-Smads), namely Smad-2, and Smad-3 proteins [11]. These activated R-Smads heterodimerize with the Co-Smad, mainly Smad-4, to build up a transcriptionally active complex which translocates to the nucleus where it modulates the expression of TGF-β target genes [12].

Recently, it is believed that diabetic nephropathy is one kind of chronic inflammation [13]. Growing evidences demonstrated that activation of nuclear factor-kappa B (NF-κB) and subsequently coordinated expression of gene products may play an important role in the pathogenesis of diabetic nephropathy [14]. Mononuclear macrophage infiltration and abnormal expression of inflammatory mediators including intercellular adhesion molecule-1 (ICAM-1), MCP-1, TGF-β1, can be observed in nephridial tissue in the early stages of diabetic nephropathy [15]. In diabetic setting, the activated NF-κB translocates into the nucleus and triggers the expression of its target genes including ICAM-1, MCP-1, and TGF-β1 which in turn induce persistent and enhanced inflammation, and finally lead to excessive fibronectin (FN) production and ECM accumulation resulting in acceleration of the pathogenesis of glomerular sclerosis and tubulointerstitial fibrosis [16].

Oryeongsan (known as Wulingsan in China) is a well-known blended traditional herbal medicine, comprising 5 herbs, *Alisma orientale (Sam.) Juz*. (Alismataceae), *Polyporus umbellatus* Fries (Polyporaceae), *Atractylodes macrocephala* Koidez (Compositae), *Poria cocos* Wolf (Polyporaceae) and *Cinnamomum Cassia* Presl (Laruaceae). It was originally recorded in an ancient Chinese medicine book “Treatise on Febrile Diseases” (Shanghan Lun or Shanghan Zabing Lun in Chinese) and has been reported to possess renal protective effects from renal diseases such as diabetes induced renal damage [17], and adriamycin-induced nephrotic syndrome [18] in experimental models.

An important question is whether Oryeongsan would have an effect on HG-induced mesangial cell fibrogenesis. Therefore, the present study was performed to determine the possible effects of a crude water extract of Oryeongsan on proliferative, inflammatory and fibrogenic phenotypic changes of primary rat mesangial cells induced by HG.

## Methods

### Preparation of a water extract from Oryeongsan

Herbarium voucher specimen of Oryeongsan (No. HBH112) was kindly provided from Korea Institute of Oriental Medicine, Daejeon, South Korea. Formula of Oryeongsan, *Alisma orientale* (Sam.) Juz*.* (Alismataceae), *Polyporus umbellatus* Fries (Polyporaceae)*, Atractylodes macrocephala* Koidez (Compositae), *Poria cocos* Wolf (Polyporaceae) *and Cinnamomum Cassia* Presl (Laruaceae) were mixed according to the ratio of 5:3:3:3:1 in weight respectively and ground into a crude powder. Oryeongsan (281 g) was boiled with 2 L of distilled water at 100°C for 2 h. The extract was filtered through Whatman No. 3 filter paper and centrifuged at 990 × g for 20 min at 4°C. Supernatant was concentrated using a rotary evaporator and then the resulting extract (65.67 g) was lyophilized using a freeze-drier and retained at −70°C until required.

### Mesangial cell cultures

All experimental procedures were carried out in accordance with the National Institute of Health Guide for the Care and Use of Laboratory Animals and were approved by the Institutional Animal Care and Utilization Committee for Medical Science of Wonkwang University (No.WKU12-14). Rat mesangial cells were isolated and cultured by modifying a standard collagenase digestion method as previously described [19]. Briefly, male Sprague–Dawley (SD) rats weighing 150–175 g were anesthetized and their kidneys removed. Renal cortical tissues were separated from the medulla and minced in D-Hank’s balanced buffer using sterile conditions. Minced renal cortical tissues were filtered through 220, 100, and then 76 mm stainless steel mesh filters and subsequently digested in 0.1% collagenase (type IV) solution at 37°C for 30 min. After centrifuging at 1,000 rpm/min for 10 min at room temperature, pellets were re-suspended with 5.4 mmol/L glucose DMEM supplemented with 15% FBS, 100 U/mL penicillin, 100 mg/ml streptomycin, and 5 mg/ml bovine insulin. The dispersed glomeruli were placed in 100 mm plastic dishes with the same culture medium and incubated in a humidified incubator at 37°C under 95% air and 5% CO_2_. The culture medium was changed every 3 days. Cell outgrowth from glomeruli was observed every 2–3 days after seeding, which would reach confluence after 30 days. The cells from passages 5–10 were employed in the current study. In some experiments, the TGF-β type І receptor inhibitor SB431542 (Sigma, 10 μM) was used to test TGF-β type І –independent mesangial fibrosis and inflammation.

### Assessment of cell number

Rat mesangial cells were plated in culture flasks and incubated with indicated concentrations of Oryeongsan (from 0, 1, 10, and 50 *μ*g/mL) with or without HG (25 mM) for 24 h. The cells were removed by treatment of trypsine/EDTA solution and collected by centrifugation. Resuspend the cell pellet in 1 ml medium, moved in different tube to resuspension of 10 μl and then mixed with 0.4% trypan blue. The mixture of 10 μl was added to the chamber ports on one side of the Countess™ cell counting chamber slide according to Invitrogen Corporation’s recommended protocol using a Countess™ Automated Cell Counter (Invitrogen Corporation, Van Allen Way, Carlsbad, CA).

### Measurement of cell proliferation

[^3^H]-thymidine incorporation was measured to determine the effect on rat mesnagial cell proliferation. Quiescent cells were treated with 25 mM glucose and Oryeongsan, respectively, and 1 μCi of [^3^H]-thymidine was added (methyl-[^3^H] thymidine 50 Ci/mmol; Amersham, Oakville, Ontario, Canada). After incubation for 24 h, cells were washed once with 2 ml of ice-cold PBS for 10 minutes, extracted three times with 2 ml of cold 10% TCA for 5 minutes each time, and solubilized for at least 30 minutes at room temperature in 0.2 ml 0.3 N NaOH, 1% SDS. After neutralizing with 0.2 ml 0.3 N HCI, [^3^H]-thymidine activity was measured in a liquid scintillation counter (Beckman LS 7500, Fullerton, CA). Each experiment was performed in triplicate or quadruplicate.

### Western blot analysis

Cell homogenates (40 μg of protein) were separated on 10% SDS-polyacrylamide gel electrophoresis and transferred to nitrocellulose paper. Blots were then washed with H_2_O, blocked with 5% skimmed milk powder in TBST [10 mM Tris–HCl (pH 7.6), 150 mM NaCl, 0.05% Tween-20] for 1 h and incubated with the appropriate primary antibody at dilutions recommended by the supplier. Then the membrane was washed, and primary antibodies were detected with goat anti-rabbit-IgG conjugated to horseradish peroxidase, and the bands were visualized with enhanced chemiluminescence (Amersham, Buckinghamshire, UK). Protein expression levels were determined by analyzing the signals captured on the nitrocellulose membranes using the Chemi-doc image analyzer (Bio-Rad, Hercules, CA).

### RNA isolation and real-time qRT-PCR

A kit from Qiagene (RNeasy™ Plus mini kit) was used for RNA isolation from cell cultures, and RNA quality was tested by measuring the ratio 260/280 nm in a UV-spectrophotometer. Real-time quantitative RT-PCR analysis was carried out in a 48-well plate using the Opticon MJ Research instrument (Bio-rad Inc) and optimized standard SYBR Green 2-step qRT-PCR kit protocol (DyNAmo™, Finnzymes, Finland). Specific sense and antisense primers used were as follows respectively: ICAM-1, sense: 5′-GCT GCT ACC ACA CTG ATG ACG ACA A-3, anti-sense: 5′-CAG TGA CCA TCT ACA GCT TTC CGG-3′; MCP-1, sense: 5′-GAT CTC AGT GCA GAG GCT CG-3′, anti-sense: 5′-TGC TTG TCC AGG TGG TCC AT-3′ [20]; type IV collagen, sense: 5′-GGT GTT GCA GGA GTG CCA G-3′, anti-sense: 5′-GCA AGT CGA AAT AAA ACT CAC CAG-3′; CTGF, sense: 5′-GCA AAT AGC CTG TCA ATC TC-3′, anti-sense: 5′-TCC ATA AAA ATC TGG CTT GT-3′; TGF-β1, sense: 5′-CAA CAA TTC CTG GCG TTA CCT TGG-3′, anti-sense: 5′-GAA AGC CCT GTA TTC CGT CTC CTT-3′ [21]; GAPDH, sense: 5′-CGA GAA TGG GAA GCT TGT CAT C-3′, anti-sense: 5′-CGG CCT CAC CCC ATT TG-3′. The PCR was started at 95°C for 15 minutes (hot start) to activate the AmpliTaq polymerase, followed by a 45-cycle amplification (Denaturation at 94°C for 20 seconds, annealing at 60°C for 30 seconds, extension at 72°C for 60 seconds, and plate reading at 60°C for 10 seconds). The temperature of PCR products was elevated from 65°C to 95°C at a rate of 0.2°C/1 sec, and the resulting data were analyzed by using the software provided by the manufacturer.

### Gelatin zymography

MMP-2 and MMP-9 enzymatic activities were assayed by gelatin zymography [22]. Samples were electrophoresed on 1 mg/ml gelatin containing 10% SDS-polyacrylamide gel. After electrophoresis, the gel was washed twice with washing buffer (50 mM Tris–HCl, pH 7.5, 100 mM NaCl, 2.5% Triton X-100), followed by a brief rinsing in washing buffer without Triton X-100. The gel was incubated with incubation buffer (50 mM Tris–HCl, pH 7.5, 150 mM NaCl, 10 mM CaCl_2_, 0.02% NaN_3_, 1 μM ZnCl_2_) at 37°C. After incubation, the gel was stained with Commassie brilliant blue R-250 and destained. A clear zone of gelatin digestion was represented with the MMP activity.

### Transient transfection and luciferase reporter assay

Mesangial cells were grown to 60–80% confluence, and the cells were transiently co-transfected with the plasmids using Lipofectamine LTX (Invitrogen, Carlsbad, CA) according to the manufacture’s protocol. Briefly, plasmids linked to a luciferase reporter (MMP-2 promoter) were kindly provided from Lee ST, Yonsei University, Seoul, Republic of Korea. Cells were transiently transfected with 0.5 μg of β-galactosidase (β-gal). The transfected mixture containing 0.5 μM of either the reporter gene constructs (β-gal) was mixed with the Lipofectamine LTX reagent and added to the cells. After 24 h, the cells were treated with Oryeongsan for 30 min and stimulated with 25 mM glucose for 24 h and then lysed. The luciferase and β-gal activities were determined as described elsewhere using luciferase assay kit (Promega, Madison, WI). The luciferase activities were normalized with respect to the β-gal activity.

### Preparation of cytoplasmic and nuclear extracts

The cells were rapidly harvested by sedimentation and nuclear and cytoplasmic extracts were prepared on ice as previously described by the method [23]. Cells were harvested and washed with 1 ml buffer A (10 mM HEPES, pH 7.9, 1.5 mM MgCl_2_, 19 mM KCl) for 5 min at 600 × g. The cells were then resuspended in buffer A and 0.1% NP 40, left for 10 min on ice to lyse the cells and then centrifuged at 600 × g for 3 min. The supernatant was saved as cytosolic extract. The nuclear pellet was then washed in 1 ml buffer A at 4,200 × g for 3 min, resuspended in 30 μl buffer C (20 mM HEPES, pH 7.9, 25% glycerol, 0.42 M NaCl, 1.5 mM MgCl2, 0.2 mM EDTA), rotated for 30 min at 4°C, then centrifuged at 14,300 × g for 20 min. The supernatant was used as nucleus extract.

### Immunofluorescence microscopy

Rat mesangial cells on glass coverslips were fixed in 4% paraformaldehyde for 30 minutes and then permeabilized with 0.4% Triton X-100 for 5 minutes in PBS, washed 3 times with PBS. After blocking in 1% BSA, samples were incubated with primary antibody (p-Smad-2, and Smad-4) at 4°C overnight. Corresponding secondary antibodies were labeled with Alexa Fluor 488 (1:200; Molecular Probes, Eugene, OR). Rat mesangial cells nuclei were counterstained with DAPI. Recording and analysis of fluorescence signals were performed using ImagePro software 5.0 (Media Cybernetics, Inc., MD).

### Statistical analysis

All the experiments were repeated at least three times. The results were expressed as a mean ± S.E., and the data were analyzed using one-way ANOVA followed by Student’s *t*-test to determine any significant differences. P < 0.05 was considered as statistically significant.

## Results

### Effect of Oryeongsan on HG-induced mesangial cell proliferation

**Figure 1:**
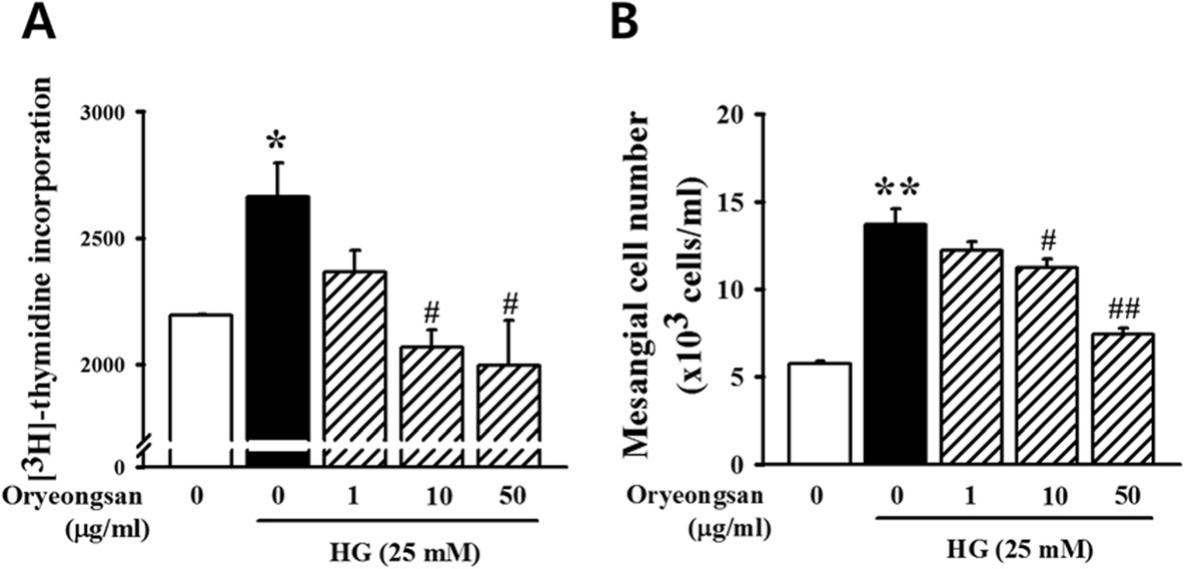
**Effects of Oryeongsan on rat mesangial cell proliferation. (A)** Mesangial cells were seeded into the 24-well plates. After confluence, the cells incubated for 24 h with or without HG and various concentrations of Oryeongsan, and then pulse-labeled with [^3^H]-thymidine for 24 h. **(B)** Inhibitory effect of Oryeongsan on proliferation of mesangial cells. Cells were incubated with indicated concentrations of ORS (from 0, 1, 10, and 50 *μ*g/mL) with or without HG (25 mM) for 24 h. The proliferation inhibition was determined by used Countess™ cell counting. Results are expressed as the mean ± S.E. from five independent experiments. *p < 0.05 vs. control; #p <0.05 vs. HG alone.

In the [^3^H]-thymidine incorporation assay (Figure 1A), stimulation with HG (25 mM) increased cell proliferation. Oryeongsan (1–50 μg/ml) inhibited HG-induced cell proliferation in a dose-dependent manner. HG-induced increase of cell number also reduced by pretreatment of Oryeongsan (Figure 1B).

### Effect of Oryeongsan on HG-induced mesangial fibrosis

**Figure 2:**
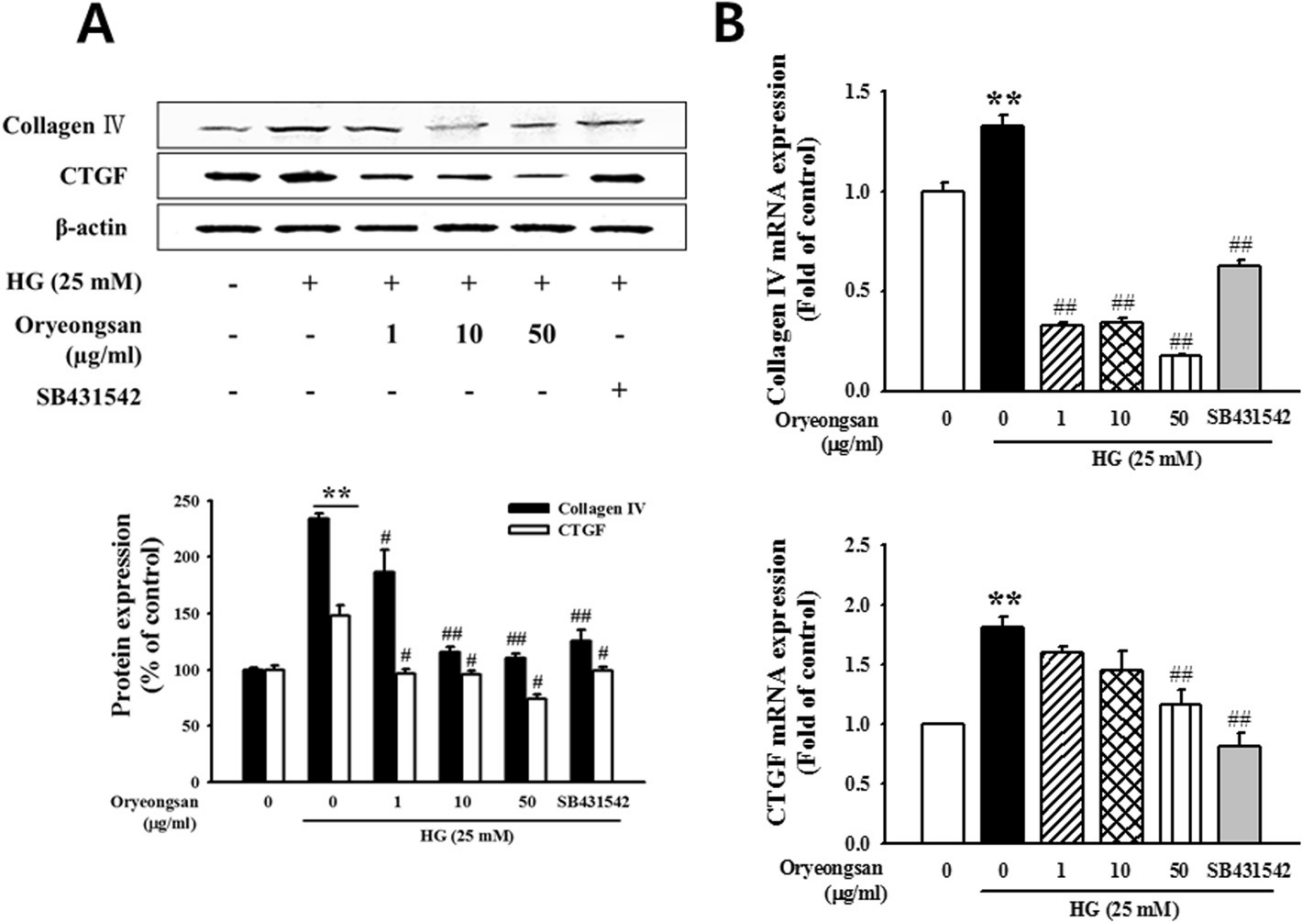
**Effect of Oryeongsan on HG-induced type IV collagen and CTGF level.** Cell lysates were used for Western blot analysis with a primary antibody against type IV collagen and CTGF. β-actin protein was used as an internal control. **(A)** Western blotting and **(B)** real-time PCR showed protein and mRNA levels of type IV collagen and CTGF in Oryeongsan-treated and HG-stimulated rat mesangial cells. Each value represents the means ± S.E. of five independent experiments. **p < 0.01 vs. control; #p <0.05, ##p < 0.01 vs. HG alone.

This study evaluated whether HG stimulated mesangial cell fibrogenesis and whether Oryeongsan reversed it. Mesangial matrix accumulation was shown to be responsible for renal fibrosis [24]. To investigate inhibitory effects of Oryeongsan on HG-instigated mesangial matrix expansion, production of collagen IV and CTGF was examined. HG-elevated fibrogenic collagen IV protein expressions, was attenuated by adding ≥10 μg/ml Oryeongsan. In addition, HG-augmented production of CTGF was suppressed in Oryeongsan-treated rat mesangial cell (Figure 2A). The realtime RT-PCR analysis were employed to confirm that Oryeongsan regulate HG-triggered induction of mesangial type IV collagen and CTGF at transcriptional levels. As shown in Figure 2B, the mRNA expression of type IV collagen and CTGF were markedly reduced by Oryeongsan in HG-exposed cells. Collagen IV secretion encumbered by Oryeongsan was most likely attributed to decreased CTGF production due to its supplementation. Of interest, SB431542, a TGF-beta type I receptor inhibitor, inhibited collagen IV and CTGF levels under high glucose condition. Thus, these results suggest that Oryeongsan improved HG stimulated mesangial cell fibrogenesis, and TGF-β signal play a part in HG-induced collagen IV and CTGF expression.

### Effect of Oryeongsan on MMP dysfunction initiated by HG

**Figure 3:**
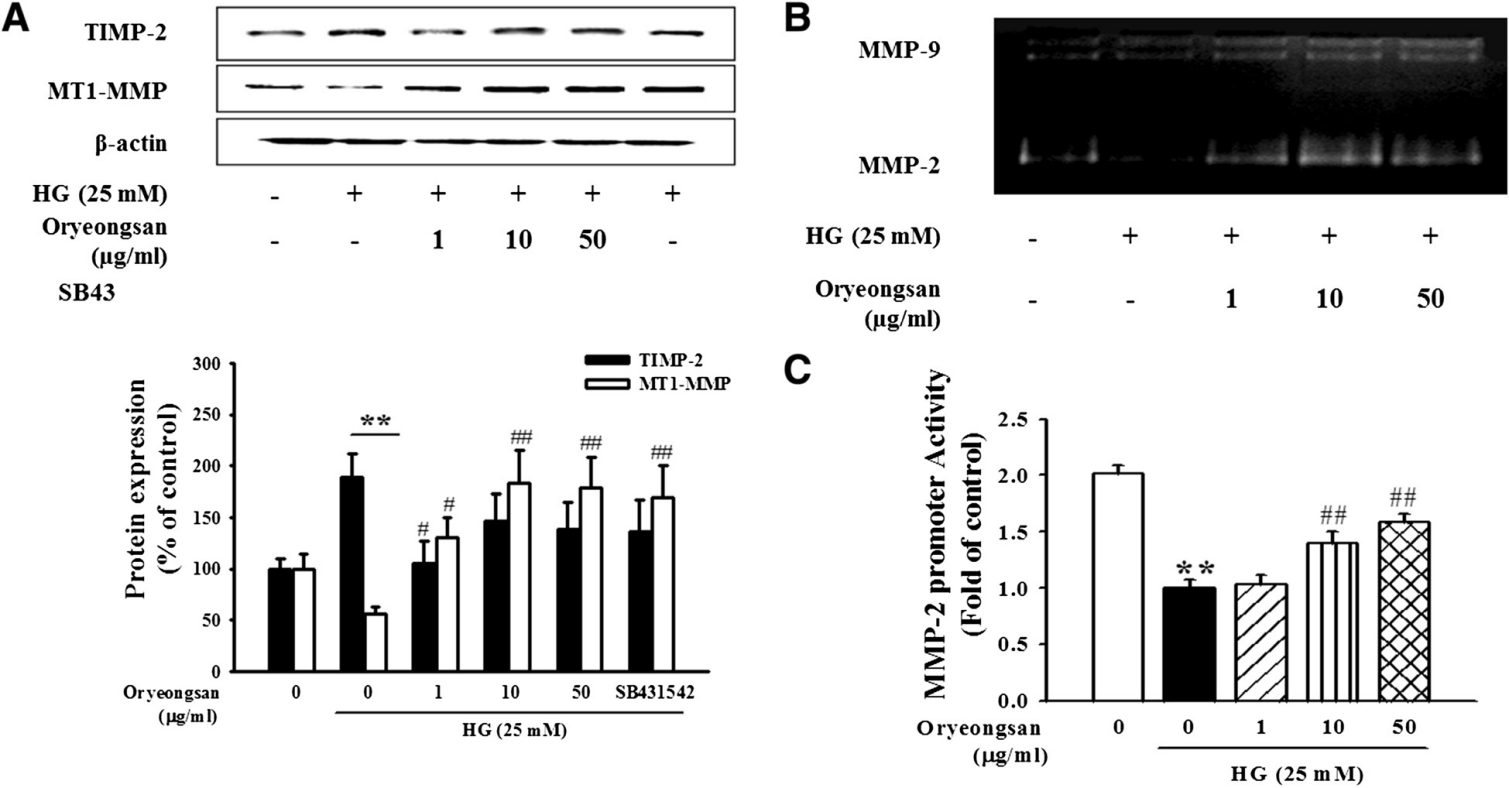
**Effect of Oryeongsan on HG-modulated expression of MMP-2, MT-1 MMP and TIMP-2. (A)** Western blot data showing expression of TIMP-2 and MT-MMP-1 in rat mesangial cells treated with Oryeongsan under HG concentrations. β-actin protein was used as an internal control. **(B)** For gelatin zymography measuring MMP-2 activity, collected culture media were run for electrophoresis on 7.5% SDS-PAGE copolymerized with 0.1% gelatin as the substrate. **(C)** Effect of Oryeongsan on MMP-2 promoter activity in rat mesangial cell. This was followed by harvesting and determining their luciferase, and β-gal activities were determine. The luciferase activities are expresses relative to the fold of HG and are the mean ± S.E. of five independent experiments. **p < 0.01 vs. control; ##p <0.01 vs. HG alone. The bar graphs (mean ± S.E., n = 3) in bottom panel represent quantitative results obtained from a densitometer. Values not sharing a letter are significantly different as P < 0.05.

MT-1 MMP is known to activate pro-MMP-2 to its active form, thereby degrading ECM [25]. Expression of MT-1 MMP and TIMP-2 in HG-treated mesangial cell was assessed using Western blot analysis. Treatment of Oryeongsan was increased on HG-reduced MT-1 MMP expression. Whereas, TIMP-2 expression was augmented in cells exposed to HG, and decreased by Oryeongsan (Figure 3A). Similar to pretreatment of Oryeongsan, SB431542 was increased MT1-MMP protein expression compared with HG alone. The ECM-degrading activity of MMP-2 was examined by using gelatin zymography assay. HG suppressed conversion of proMMP-2 to its active form. However, Oryeongsan substantially enhanced gelatinolytic MMP-2 activity diminished by HG (Figure 3B). In addition, Oryeongsan enhanced MMP-2 promotor activity diminished by HG (Figure 3C). Accordingly, compelling evidence was drawn from this study that Oryeongsan had the potential capability to block HG-induced glomerulosclerosis and renal fibrosis.

### Interruption of HG-induced TGF-β/smad signal pathway by Oryeongsan

**Figure 4:**
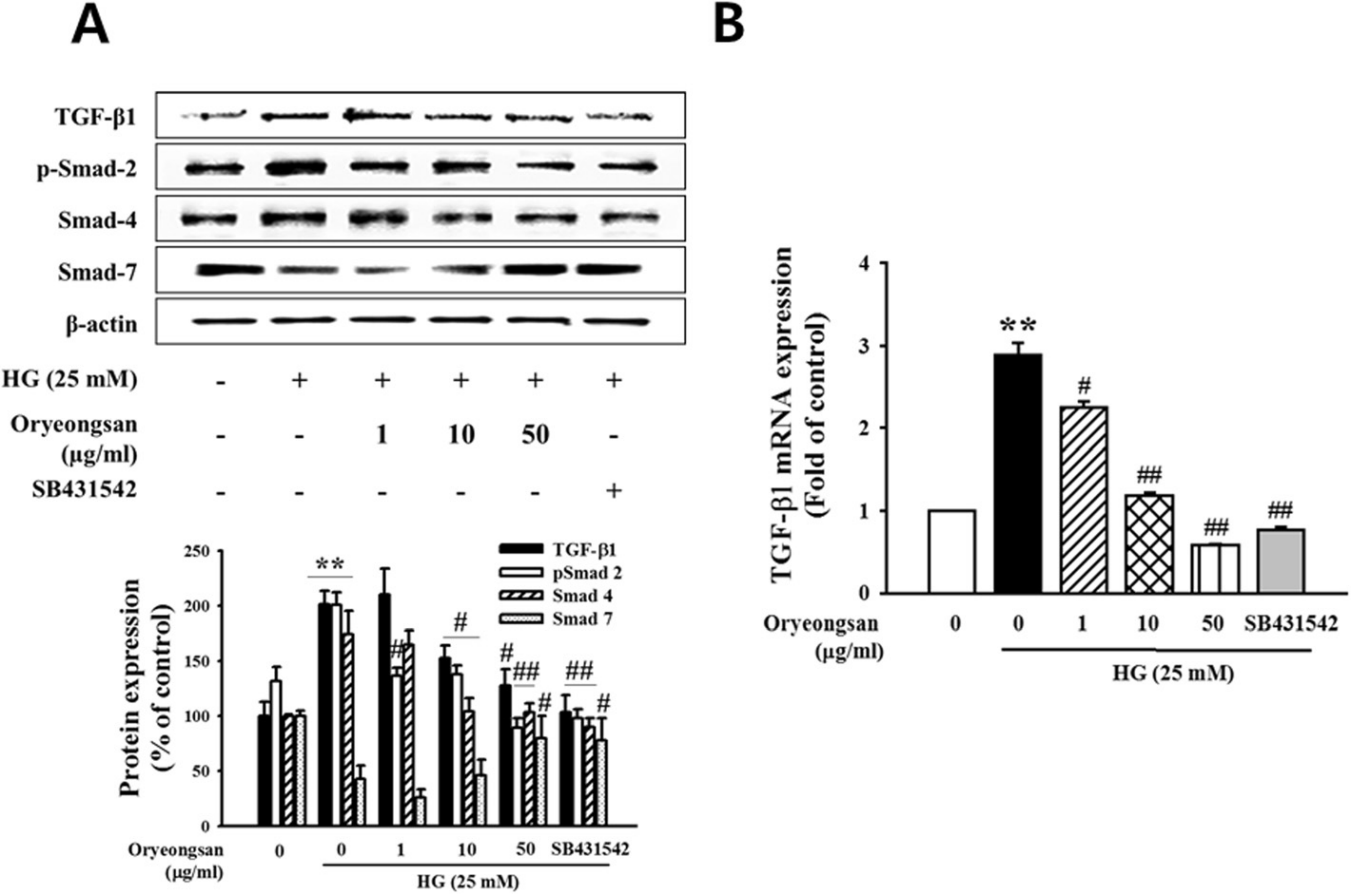
**Effect of Oryeongsan on the relative levels of TGF-β** **1 and Smads. (A)** The protein bands detected by western blotting, β-actin was used as the internal standard in each sample. **(B)** real-time PCR showing mRNA levels of TGF-β in Oryeongsan-treated and HG-stimulated rat mesangial cells. Each value represents the means ± S.E. of five independent experiments. **p < 0.01 vs. control; #p <0.05, ##p < 0.01 vs. HG alone.

**Figure 5:**
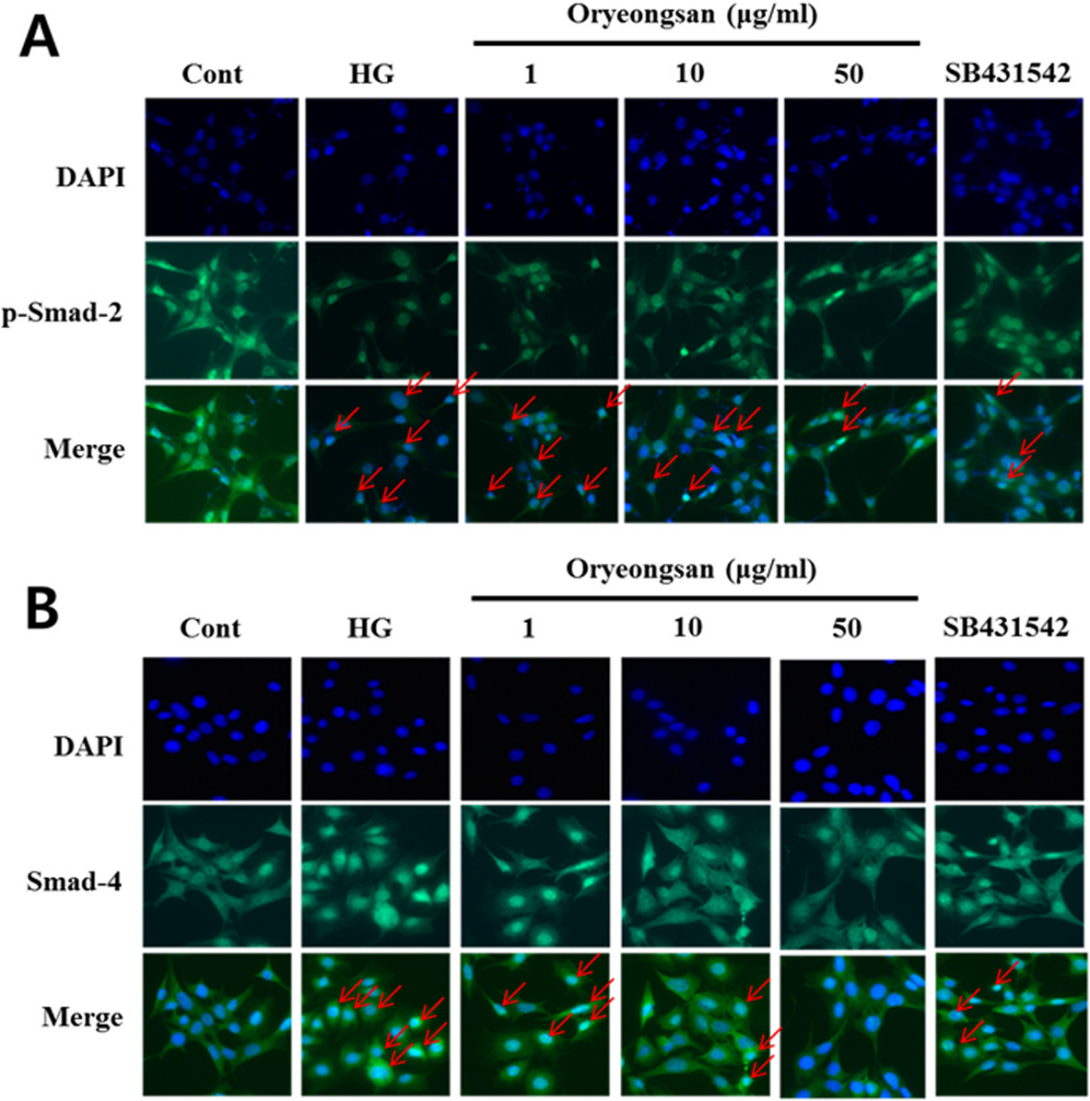
**Effects of Oryeongsan on translocation of p-Smad-2 (A) and Smad-4 (B) into the cell nucleus in rat mesangial cells.** Immunofluorescent images of p-Smad-2 **(A)** and Smad-4 **(B)** nuclear translocation (red arrow) under the laser scanning confocal microscopy were show (magnification. 400X). Nuclei were stained with DAPI (blue) and p-smad-2 **(A)** and Smad-4 **(B)** were stained with Alexa Fluor 488 (green). Green fluorescence indicates localization of p-Smad-2/Smad-4. Respective data were obtained from five independent experiments.

TGF-β has been described to contribute a critical role in causing glomerulosclerosis and renal fibrosis. The signaling pathway activated by TGF-β1 involves type I receptor-mediated phosphorylation of Smad-2 and Smad-3, thereby allowing them to associate with Smad-4 and to translocate into the nucleus for gene expression [26]. HG enhanced cellular levels of TGF-β, phospho-Smad-2 and Smad-4 at 24 h after stimulation (Figure 4A). When mesangial cell was treated with Oryeongsan, Oryeongsan was decreased TGF-β1 expression and Smad-2 phosphorylation (Figure 4A). Whereas, Smad-7 (I-Smad) expression increased by Oryeongsan under HG condition. In addition, Oryeongsan was reduced HG-induced TGF-β1 mRNA expression (Figure 4B). As shown in Figure 5A and B, p-Smad-2 could bind to Smad-4 and was translocated into the nucleus in HG condition. Staining intensities of p-Smad-2/Smad-4 of the HG condition highly increased and p-Smad-2/Smad-4 expression levels in nuclear extracts markedly decreased when treated Oryeongsan. TGF-β acts through the Smad pathway involving receptor-regulated Smad-2 and common-mediated Smad-4 [27]. Thus, disturbing Smad-2 phosphorylation or Smad-4 expression may be an approach that will terminate TGF-β signaling, thereby blocking TGF-β-mediated fibrosis. Furthermore, as shown in Figures 4 and 5, Oryeongsan down regulated TGF-β and Smads level in the same league as TGF-β type І receptor inhibitor SB431542.

### Attenuation of inflammatory ICAM-1 and MCP-1 by Oryeongsan in HG-stimulated mesangial cell

**Figure 6:**
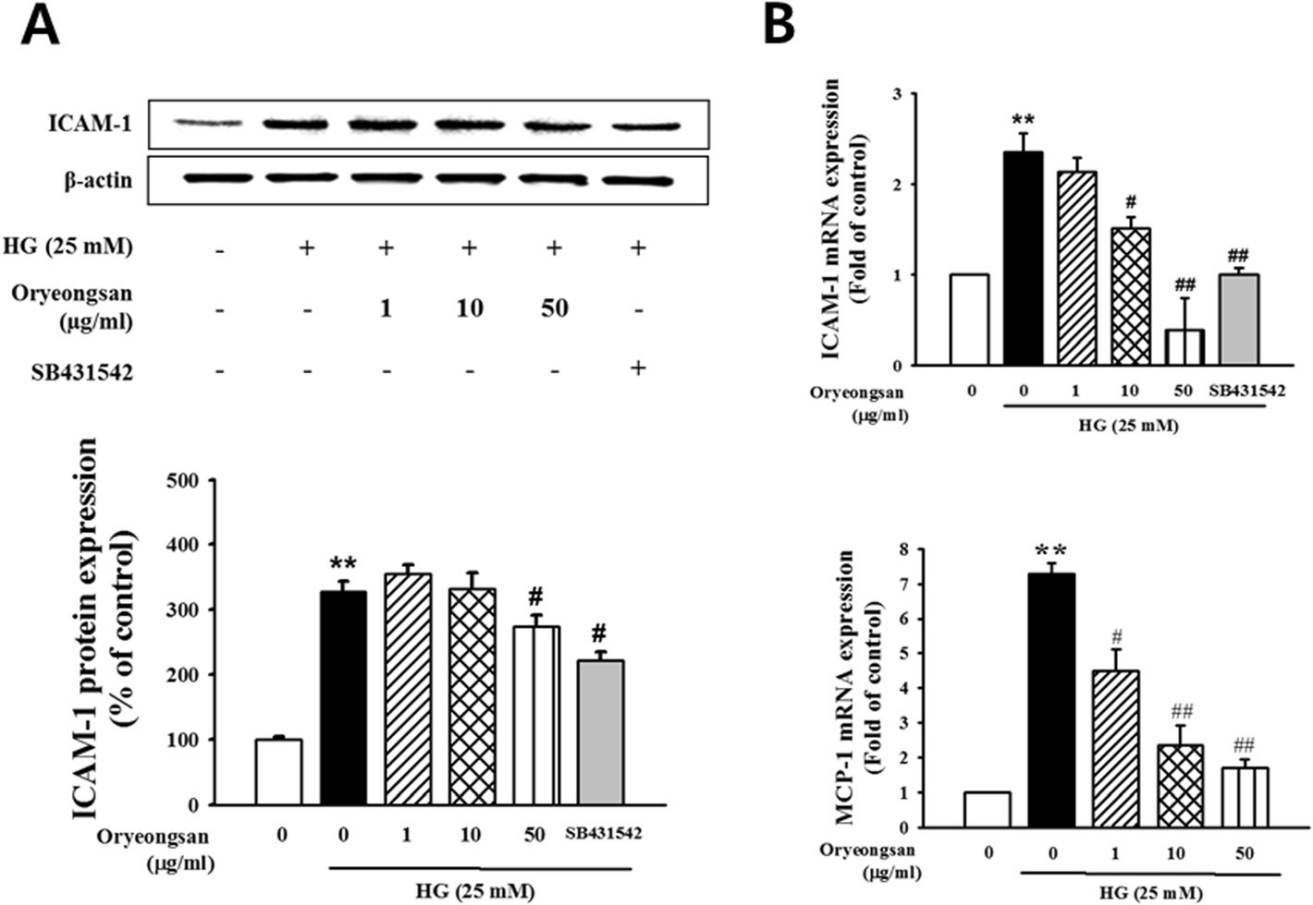
**Effect of Oryeongsan-induced inflammatory ICAM-1 and MCP-1. (A)** Cell lysates were used for Western blot analysis with a primary antibody against ICAM-1. β-actin protein was used as an internal control. **(B)** real-time PCR showing mRNA levels of ICAM-1 and MCP-1 in Oryeongsan-treated and HG-stimulated rat mesangial cells. Each value represents the means ± S.E. of five independent experiments. **p < 0.01 vs. control; #p <0.05, ##p < 0.01 vs. HG alone.

This study elucidated that Oryeongsan may alleviate mesangial inflammation possibly involved in renal fibrotic process. ICAM-1, an important adhesion molecule related to vascular inflammation, promotes inflammatory cells including mononuclear macrophage infiltration into glomeruli and renal interstitium and accelerates glomerular sclerosis in diabetes [28]. This study elucidated that Oryeongsan may alleviate mesangial inflammation possibly involved in renal fibrotic process. HG enhanced ICAM-1 protein expression (Figure 6A). In addition, ICAM-1 and MCP-1 mRNA level also were enhanced in HG-exposed mesangial cells, evidenced by real-time PCR (Figure 6B). This elevation was markedly attenuated by treated by 50 μg/ml Oryeongsan and SB431542, TGF-β type І receptor inhibitor. This implies that TGF-β was an upstream regulator of pro-inflammatory ICAM-1 and MCP-1. It seems to be plausible that there was a crosstalk between mesangial fibrosis and inflammation that was disrupted by Oryeongsan. It seems to be plausible that there was a crosstalk between mesangial fibrosis and inflammation that was disrupted by Oryeongsan.

### Blockade of NF-κB activity by Oryeongsan

**Figure 7:**
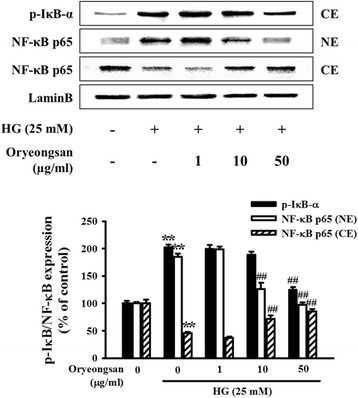
**Blockade of nuclear translocation of NF-**κ**B in rat mesangial cells.** Confluent cells were incubated with 1–50 μg/ml Oryeongsan for 24 h under the conditions of HG. Western blot analysis with a primary antibody against NF-κB/p-IκB-α, and cytoplasmic (CE) and nuclear (NE) fractions were obtained. Respective blot data were obtained from five independent experiments.

NF-κB is activated, it translocates into the nucleus and binds to a specific DNA sequence on target gene promoter to trigger the transcription of target genes related to inflammatory injury [29], which promote mesangial cells proliferation and mononuclear macrophage infiltration and eventually accelerate glomerularsclerosis in diabetic kidney. In Western analysis, HG caused NF-κB p65 translocation in the nucleus, which is inhibited by pretreatment with Oryeongsan. In addition, HG-induced phosphorylation of IκB-α, the inhibitors of NF-κB activity, was reduced by Oryeongsan in the cytoplasm (Figure 7).

## Discussion

In the current study, we showed an inhibitory effect of Oryeongsan on HG-induced rat mesangial cell fibrogenesis and inflammation. We demonstrated that Oryeongsan retarded HG-induced mesangial cell fibrogenesis through disturbing TGF-β/Smad signaling. Oryeongsan suppressed HG-induced hyperplasia and ECM expansion by improving matrix-degrading MMP system involving TIMP-2. The HG-stimulated CTGF and type IV collagen induction entailed TGF-β-Smad-responsive pathways that were encumbered by Oryeongsan. Finally, Oryeongsan attenuated inflammatory ICAM-1 and MCP-1 expression in the mesangium.

Diabetic nephropathy, a progressive kidney disease caused by angiopathy of capillaries in the kidney glomeruli, is one of the most severe complications of type I and type II diabetes [30]. The earliest detectable change in the course of diabetic nephropathy is an expansion of the glomerular mesangium, which is caused by excessive accumulation of ECM proteins [31].

Glomerular mesangial cells, an important member of renal glomerulus, play a major role not only in physiological functions but also in pathogenesis of kidney diseases [32]. Mesangial cells comprise approximately one third of decapsulated glomerular cell population with endothelial cells and glomerular visceral epithelial cells comprising the rest. Mesangial cells maintain the structural architecture of the glomerular capillary similar to the function of certain microvascular pericytes. These cells also contribute to mesangial matrix homeostasis, regulate filtration surface area and phagocytose apoptotic cells or immune-complexes formed at or delivered to the glomerular capillaries. Knowledge regarding biological responses of mesangial cells and mesangial matrix in disease states is derived from careful morphological and morphometric evaluation of human kidney biopsies and kidney tissue harvested from experimental models. As such, cell hypertrophy and proliferation as well as matrix expansion were identified as few of the common biological responses of mesangial cell to injury.

CTGF are expressed in the mesangium and play an important role in the ECM-synthesizing process, while MMP is involved in inducing the enzymatic breakdown of ECM [33]. Thus, in renal diseases, targeting CTGF and MMP is considered to resolve or prevent glomerulosclerosis and interstitial fibrosis. This study evaluated whether Oryeongsan modulate HG-induced production of type IV collagen and expression of CTGF. Western blot data showed that Oryeongsan inhibited the type IV collagen and CTGF protein expression enhanced by HG. In addition, Oryeongsan improved the HG-triggered MMP system dysfunction by enhancing ECM-degrading MT-1 MMP expression and repealing TIMP-2 expression. Accordingly, compelling evidence was drawn from this study that Oryeongsan had the potential capability to block HG-induced glomerulosclerosis and renal fibrosis.

Mesangial ECM components not only provide structural support of the glomerular capillary tuft, but also play a more active role by affecting adhesion, migration and proliferation. Activation of the TGF-β1 loop leads to cell cycle arrest and ECM synthesis [34]. In mesangial cells, TGF-β1 inhibits growth and stimulates the synthesis of collagens, fibronectin, laminin and proteoglycans [35]. The Smads proteins following TGF-β1 are thought to be some of the most important factors in the process of ECM accumulation. Thereby, induction and activation of the TGF-β/Smad signaling cascade is critical for the initiation of fibrogenic cell responses. TGF-β–Smad signaling has been reported to play a vital role in the process of ECM expansion. Smad-3 and Smad-4 but not Smad-2 was required for TGF-β1-induced CTGF promoter activity and expression in osteoblasts [11]. In this study, we found that Oryeongsan disturbed the TGF-β-Smad signal pathway through retarding TGF-β expression in mesangial cells cultured by HG and downstream activation of Smad-2 and Smad-4 and enhancing the level of inhibitory Smad-7. It is shown in the present study, Oryeongsan decrease TGF-β1 and p-Smad-2/Smad-4, and increase Smad-7 expression in mesangial cells cultured by HG. In addition, p-Smad-2/Smad-4 of the HG condition highly increased and p-Smad-2/Smad-4 expression levels in nuclear extracts markedly decreased when treated Oryeongsan. These results suggest that Oryeongsan prevents renal fibrosis process by inhibition of TGF-β/Smad signaling cascade in diabetic nephropathy.

In diabetic patients with nephropathy, the levels of ICAM-1 and MCP-1 recruiting inflammatory immune cells of macrophages and granulocytes were elevated [14]. It was shown that MCP-1 appeared to be responsible for diabetic ECM accumulation and early inflammation in diabetic nephropathy pathogenesis [36]. HG enhanced ICAM-1 protein expression, whereas Oryeongsan reduced that. In addition, ICAM-1 and MCP-1 mRNA level also were enhanced in HG-exposed mesangial cells. This elevation was markedly attenuated by treated with 50 μg/ml Oryeongsan. This study elucidated that Oryeongsan may alleviate mesangial inflammation possibly involved in renal fibrotic process, further diabetic nephropathy.

There is accumulating evidence demonstrating that the diabetic nephropathy is closely related to mesangial inflammation [14]. The activation of NF-κB may play a pivotal role in the progression of renal injury by inflammatory factors in diabetes [37]. Therefore, inhibition of the NF-κB pathway may represent a new therapeutic target for diabetic nephropathy treatment. Normally, NF-κB is retained in the cytoplasm by binding to an inhibitor protein, IκB. Upon stimulation, IκB is phosphorylated and degraded, and separated from NF-κB. Then the activated NF-κB is translocated into the nucleus to active transcriptional expression of downstream genes associated with inflammatory responses [38]. This study revealed that HG caused NF-κB p65 translocation in the nucleus, which is inhibited by pretreatment with Oryeongsan. In addition, phosphorylation of IκB-α, the inhibitors of NF-κB activity, by kinases results in the degradation of IκB-α with release of NF-κB, which translocates to the nucleus where it is active in the regulation of transcription gene. Here we could not rule out the possibility that anti-oxidant activity of Oryeongsan would be involved in beneficial effect of HG-induced renal fibrosis and inflammation, because ROS/ NF-κB pathway is critical for those actions. In previous report, it is supported by that Poria cocos, anti-oxidant, inhibits HG-induced mesangial proliferation [39]. Thus next study is required to clarify the involvement of oxidative stress and anti-oxidant property of Oryeongsan in renal fibrosis using various renal cells and diabetic animal models.

## Conclusion

Oryeongsan retarded diabetes-associated renal fibrosis and mesangial inflammation through disturbing TGF-β/Smad signaling. Oryeongsan suppressed HG-inflamed hyperplasia and ECM expansion by improving matrix-degrading MMP system involving TIMP-2. The HG-stimulated CTGF and type IV collagen induction entailed TGF-β-Smad-responsive pathways that were encumbered by Oryeongsan. Furthermore, Oryeongsan attenuated inflammatory ICAM-1 expression and MCP-1 production in the mesangial cells under HG.

Taken together, these data provide the first evidence that Oryeongsan may play an important role in the prevention of renal fibrosis, inflammation and further glomerulosclerosis. Thus, Oryeongsan may provide new insights into the development of therapeutic drugs for diabetic nephropathy.

## Abbreviations

MC: Mesangial cell
HG: High-glucose
ECM: Extracellular matrix
MMPs: Matrix metalloproteinases
NF-κB: Nuclear factor-kappa B
TGF-β: Transforming growth factor β
ICAM-1: Intracellular cell adhesion molecule-1
MCP-1: Monocyte chemoattractant protein-1
MT1-MMP: Membrane type-1 matrix metalloproteinase
